# Poly-β-Hydroxybutyrate Production by the Cyanobacterium *Scytonema geitleri* Bharadwaja under Varying Environmental Conditions

**DOI:** 10.3390/biom9050198

**Published:** 2019-05-21

**Authors:** Manoj K. Singh, Pradeep K. Rai, Anuradha Rai, Surendra Singh, Jay Shankar Singh

**Affiliations:** 1 Centre of Advanced Study in Botany, Banaras Hindu University, Varanasi 221005, India; manojksinghbhu@gmail.com (M.K.S.); pkrai17@gmail.com (P.K.R.); anuradharaijnp@gmail.com (A.R.); 2 Division of Agronomy, ICAR-Indian Agricultural Research Institute, Pusa, New Delhi 110012, India; 3 Division of Plant Pathology, ICAR-Indian Agricultural Research Institute, Pusa, New Delhi 110012, India; 4 Departments of Environmental Microbiology, Babasaheb Bhimrao Ambedkar University, Lucknow 226025, India

**Keywords:** carbon source, cyanobacteria, PHB, *Scytonema geitleri*

## Abstract

The production of poly-β-hydroxybutyrate (PHB) under varying environmental conditions (pH, temperature and carbon sources) was examined in the cyanobacterium Scytonema geitleri Bharadwaja isolated from the roof-top of a building. The S. geitleri produced PHB and the production of PHB was linear with the growth of cyanobacterium. The maximum PHB production (7.12% of dry cell weight) was recorded when the cells of S. geitleri were at their stationary growth phase. The production of PHB was optimum at pH 8.5 and 30 °C, and acetate (30 mM) was the preferred carbon source.

## 1. Introduction

Synthetic polymers are potent environmental toxic pollutants which are non-degradable and have accumulated in the environment. Therefore, the efforts of scientists have been focused on alternative environmental biopolymers. Among the biodegradable polymers, polyhydroxyalkanoates (PHAs), including polyhydroxybutyrate (PHB), have been recently recognized polyesters due to their features as biodegradable thermoplastics [1].

The PHB is mostly produced by prokaryotes and accumulated intracellularly as an energy and carbon storage material [2]. Pure PHB is of biological origin and is thermoplastic, stereo-specific and biodegradable [3,4]. It has wide applications in environmental, agricultural and biomedical fields [5,6,7].

The PHB production has been reported from cyanobacteria such as *Chloroglea fritschii* [8], *Spirulina platensis* [9], *Oscillatoria limosa* and *Spirulina* sp. [10,11,12], *Synechocystis* sp. PCC 6803 [13,14], *Nostoc muscorum* [15,16], and *Aulosira fertilissima* [7]. Kamravanesh et al. [17] found that a mutant strain of *Synechocystis* sp. PCC 6714 showed 2.5-fold higher productivity than their wild type under nitrogen and phosphorus starvation. Similarily, Troschl et al. [18] produced poly-β-hydroxybutyrate from *Synechocytis* sp. CCALA192 at pilat-scale with carbon dioxide as a sole carbon source in a photobioreactor. Several microorganisms including Gram positive like *Bacillus megaterium*, *Bacillus subtilis* and *Corynebacterium glutamicum,* Gram negative bacteria like groups of *Pseudomonas* and *Alcaligenes eutrophus*, have been reported to produce different types of polyhydroxyalkanoates (PHAs) [19]. However, studies related PHB productions by cyanobacterial members under different environmental conditions are very much limited and no reports are available for *Scytonema geitleri* Bharadwaja. Further, it is unknown whether different environmental drivers, such as pH, nitrogen source, temperature, moisture, light intensity, etc., affects PHB production rate by cyanobacteria. In this study, using a temperature and desiccation tolerant cyanobacterium *S. geitleri* Bharadawaja (isolated from the roof-top), the production of PHB in response to different environmental drivers such as pH, temperature and carbon sources (sucrose and acetate), was examined. Evidence of the presence of PHB was also examined by using spectrophotometric and gas chromatographic assays.

## 2. Materials and Methods

### 2.1. Organism and Culture Conditions

The cyanobacterium *S. geitleri* Bharadwaja was isolated from the roof-top of the Botany Department, Banaras Hindu University, Varanasi, India. The organism was purified using standard microbiological procedures and identified morphologically as per Desikachary [20]. Cultures of *S. geitleri* were grown in 250 mL Erlenmeyer flasks containing 100 mL of Chu-10 medium (pH 7.5) [21] and maintained in a culture room at 28 ± 1 °C under 14:10 h light-dark rhythm. The cultures were illuminated with a combination of day and white light fluorescent lamp giving an average light intensity of 95 µmoL m^−2^ s^−1^ on the surface of the vessels.

### 2.2. Growth Measurement

The growth of *S. geitleri* was measured by measuring the increase in its chlorophyll *a* (chl *a*) content. Cyanobacterial culture (3 mL) was harvested by centrifugation and the resulting pellet was suspended in an equal volume of 80% acetone. After thorough vortexing, the suspension was kept at 4 °C for 24 h. Thereafter, it was centrifuged and absorbance of the cell-free extract was recorded at 663 nm using 80% acetone as a blank. Chl-*a* was quantified following Mackinney [22]. Oven dried cyanobacterial pellets having constant weight were used to measure the dry weight.

### 2.3. Extraction of Polyhydroxybutyrate

The cells of *S. geitleri* (28 day old) were harvested by centrifugation at 5000× *g* for 10 min and lyophilized. The lyophilized cells were digested with 4% (*w*/*v*) sodium hypochlorite (Fisher Scientific, Ottawa, ON, Canada) at 37 °C for 15 h [23]. The residual material was centrifuged at 10,000× *g* for 10 min and the residue was washed thrice with 10 mL each of water, acetone, ethanol, and ether. The resulting residue was air dried; subjected to warm chloroform extraction and filtered. The supernatant containing PHB was concentrated using a vacuum rotary evaporator, precipitated using 10 mL of ice-cold methanol and centrifuged. The precipitate thus obtained was air dried and used for further analysis of PHB [8,24].

### 2.4. Assay of Polyhydroxybutyrate

Dried PHB was digested with 10 mL concentrated H_2_SO_4_ in a water bath at 100 °C for 20 min. After cooling and thorough mixing, the absorbance of the crotonic acid (formed after H_2_SO_4_ digestion of PHB) was recorded at 235 nm using H_2_SO_4_ as a blank [25]. The presence of PHB was further confirmed spectrophotometrically by recording the absorption spectra of the sample as well as the standard poly-β-hydroxybutyric acid (Sigma-Aldrich., St. Louis, MO, USA) from 200–500 nm.

### 2.5. Quantification of Polyhydroxybutyrate

In small screw-cap glass tubes (Borosil, Mumbai, India), 1 mL of PHB solution (dissolved in chloroform) was taken and to that, 0.85 and 0.15 mL of methanol and sulfuric acid were added respectively. The tubes were placed in a thermostat-equipped water bath for 140 min at 100 °C for degradation of PHB to its constituent *p*-hydroxybutyric acid methyl esters after methanolysis. Following this, 0.5 mL of double distilled water was added to the tubes and the tubes were shaken vigorously for 1 min. The bottom layer (organic phase composed of methyl esters) was removed, transferred to a small screw-cap glass vial and stored at −70 °C for further analysis.

The PHB was analyzed using gas chromatograph (CP-3800 GC, Varian, Inc., Mitchell Drive Walnut Creek, CA, USA) fitted with a flame ionization detector (FID). The sample (2 µL) was injected by a Hamilton syringe into a CP-Sil 8 CB column (5% diphenyl and 95% dimethyl polysiloxane, 30 m by 0.32 mm inner diameter, 1µm film thickness). The column temperature was maintained initially at 80 °C for 2 min followed by subsequent increment of 10 °C in temperature up to 200 °C for another 2 min, and finally maintained at 250 °C for 10 min. The injector and detector temperatures were kept at 200 and 250 °C respectively. While N_2_ was used as carrier gas with a flow rate of 30 mL min^−1^, H_2_ and O_2_ were, however, used as flame gases with a flow rate of 30 mL min^−1^ and 300 mL min^−1^ respectively under split mode (30:100). PHB was identified using standard poly 3-hydroxybutyric acid (Sigma-Aldrich, USA) following the method of Brandl et al. [26].

## 3. Results and Discussion

**Figure 1:**
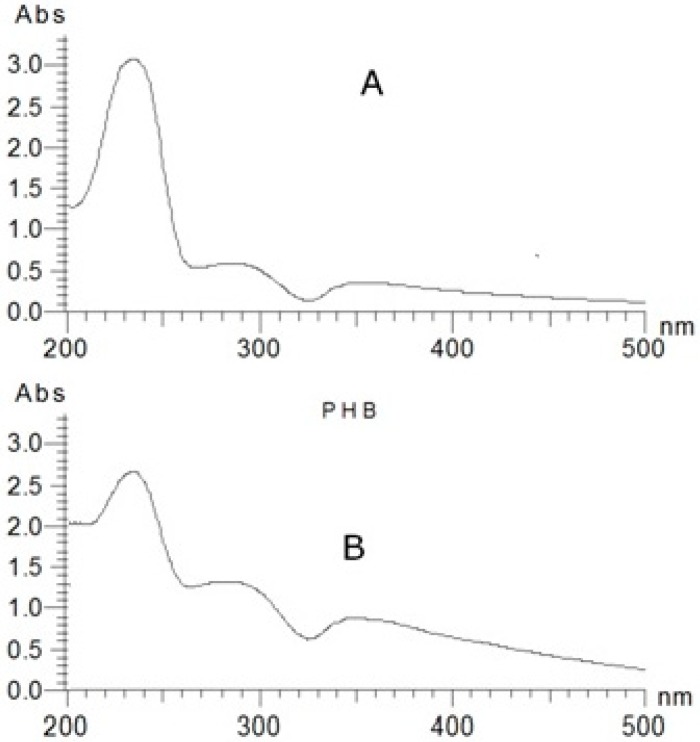
Crotonic acid peak (at 235 nm) of standard poly-β-hydroxybutyric acid (**A**) and polyhydroxybutyrate (PHB) isolated from Scytonema geitleri (**B**).

For the extraction of PHB, 28-day-old *S. geitleri* were used. A tough, translucent and grey film of PHB was recovered from the cyanobacterium. To ascertain whether the synthesized product was PHB or not, the synthesized product and standard poly 3-hydroxybutyric acid were digested with concentrated H_2_SO_4_ for their conversion to crotonic acid and their respective absorption spectra were recorded from 200–500 nm. Interestingly, both the synthesized product and standard poly 3-hydroxybutyric acid exhibited similar crotonic acid peaks with absorption maxima at 235 nm (Figure 1), thus confirming the synthesis and production of PHB in *S. geitleri*. Further, to have more conclusive evidence regarding the synthesis of PHB by *S. geitleri*, the degradation products of PHB of *S. geitleri* and standard poly 3-hydroxybutyric acid after methanolysis (*p*-hydroxybutyric acid methyl esters) were analyzed and quantified using gas chromatograph (data not shown). The appearance of the peaks at the retention time of 7.33 min for both PHB of *S. geitleri* and standard poly 3-hydroxybutyric acid again confirmed the synthesis of PHB by *S. geitleri*. Synthesis of PHB in microbial biomass has also been detected using gas chromatograph [26,27,28].

### 3.1. Relationship between Growth and Polyhydroxybutyrate Production

**Figure 2:**
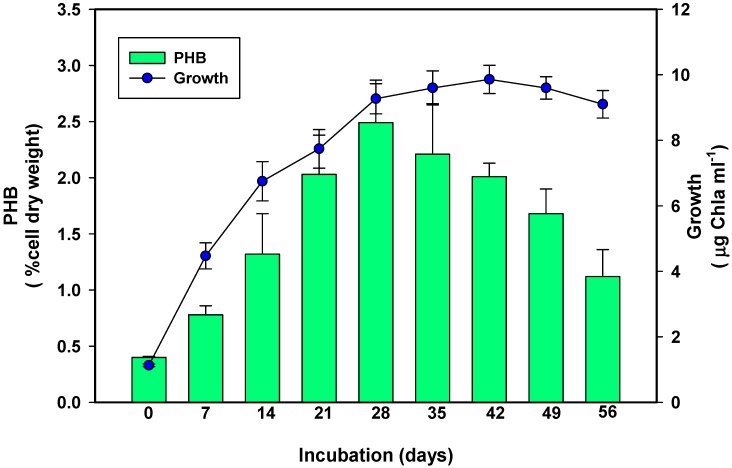
Production of PHB in *S. geitleri* in relation to growth and the incubation period. Values are the mean (*n* = 3) ± standard deviation (SD).

To ascertain whether any relationship exists between growth of *S. geitleri* and its PHB production, both the activities were measured simultaneously up to 56 days (Figure 2). It is evident from the data of Figure 2 that *S. geitleri* started producing PHB at the early growth phase and attained its optimal level (≈2.7% cell dry weight) on the 28th day of incubation. However, further increases in the incubation period resulted in a significant decrease in PHB production, attaining its minimum level on the 56th day of incubation. Similar results were also found by Ansari and Fatma [29] where they found that in *Nostoc muscorum* NCCU-442, PHB accumulation started at the early growth stage while its maximum level was recorded on the 21st day of incubation. The above contention was also supported by the observations made for other cyanobacteria [30,31,32]. PHB serves as carbon reserve and energy source for cyanobacteria [33]. The production of PHB by *S. geitleri* at its early growth phase and decrease in its production due to prolonged incubation may be explained by the facts that at early growth phases when nutrients were available in abundance and not limiting the growth medium, the cells of *S. geitleri* accumulated excess carbon in the form of PHB. However, during prolonged incubation of the cells, when the nutrients were exhausted and the resources became limiting, the cells of *S. geitleri* used PHB as an energy and carbon source to maintain its metabolic activities.

### 3.2. Relative Contribution of Carbon Sources on Polyhydroxybutyrate Production

**Figure 3:**
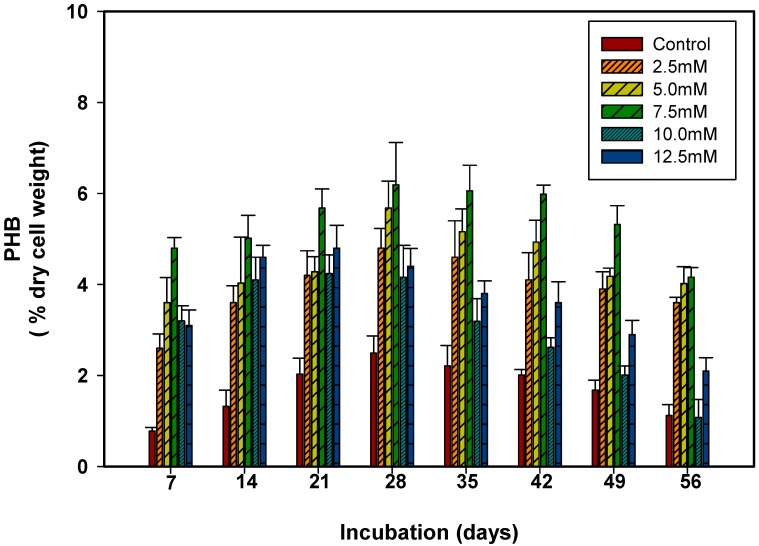
Production of PHB in *S. geitleri* in response to sucrose. Values are the mean (*n* = 3) ± SD.

**Figure 4:**
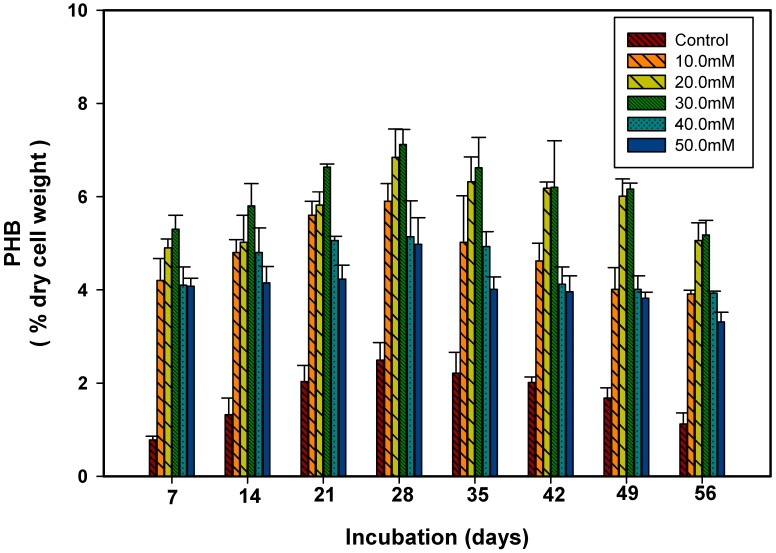
Production of PHB in *S. geitleri* in response to acetate. Values are the mean (*n* = 3) ± SD.

To ascertain the relative contribution of carbon sources (sucrose and acetate) on PHB production in *S. geitleri*, PHB content was measured in the cells of *S. geitleri* grown in the presence of sucrose (2.5–12.5 mM) and acetate (10–50 mM) for 7–56 days (Figure 3 and Figure 4). The PHB production in *S. geitleri* increased with an increase in sucrose and acetate concentrations, and attained its maximal levels of 6.19 and 7.12% of cells dry weight (cdw) at 7.5 mM sucrose and 30 mM acetate respectively on the 28th day of incubation. Similar results were also supported by Bhati et al. [34] who used the cyanobacterium *Nostoc muscoroum* Agardh. Further increases in sucrose and acetate concentrations, however, resulted in a significant decrease in PHB production. Panda et al. [35] also showed that acetate is directly used as a precursor for PHB synthesis. The PHB is synthesized in bacteria by the condensation of two moles of acetyl-CoA to acetoacetyl-CoA and subsequently to β-hydroxybutyryl-CoA [36]. The PHB synthesis is stimulated by high intracellular concentration of dihydronicotinamide adenine dinucleotide phosphate (NADPH) and a high ratio of NADPH/nicotinamide adenine dinucleotide phosphate (NADP). Many microorganisms directly utilize acetate to increase their intracellular acetyl-CoA pool for the synthesis of PHB [14,37,38]. The stimulation of PHB production in sucrose supplemented cultures of *S. geitleri* may be due to the production of NADPH necessarily required for the activity of acetoacetyl CoA reductase in PHB biosynthetic pathway [39,40]. The inhibitory effect of high concentrations of acetate and sucrose on PHB production in *S. geitleri* may be due to their high concentration causing an increase in osmotic pressure [41]. The maximum stimulation of PHB production was also reported in acetate and glucose supplemented cultures which were possibly due to the easy access to the precursor, i.e., acetate and NADPH [16,39]. Besides sucrose and acetate, other carbon and nitrogen sources have also been used to see the stimulation of PHB in cyanobacteria and bacteria [40,42]. Nishioka et al. [43] obtained 55% (dcw) PHB as carbon dioxide as the sole carbon source from the mutant strain of *Synechococcus* sp. MA-19 under phasphate limited conditions.

### 3.3. Polyhydroxybutyrate Production in Response to pH and Temperature

**Figure 5:**
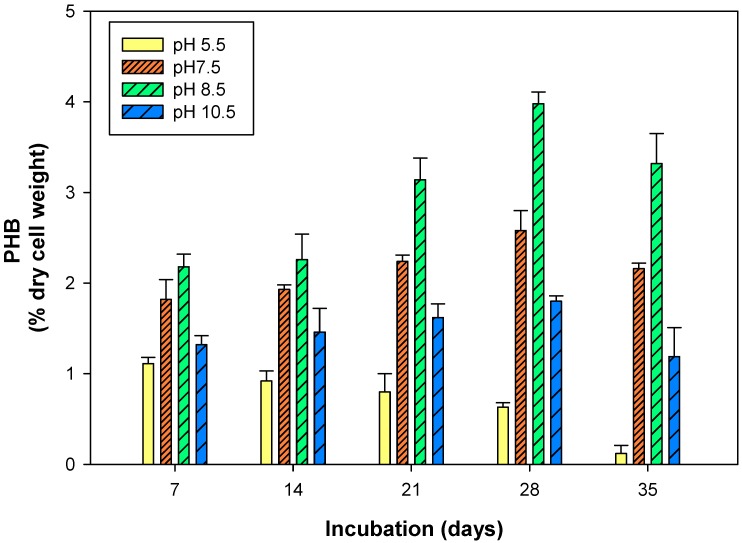
Production of PHB in *S. geitleri* in response to pH. Values are the mean (*n* = 3) ± SD.

**Figure 6:**
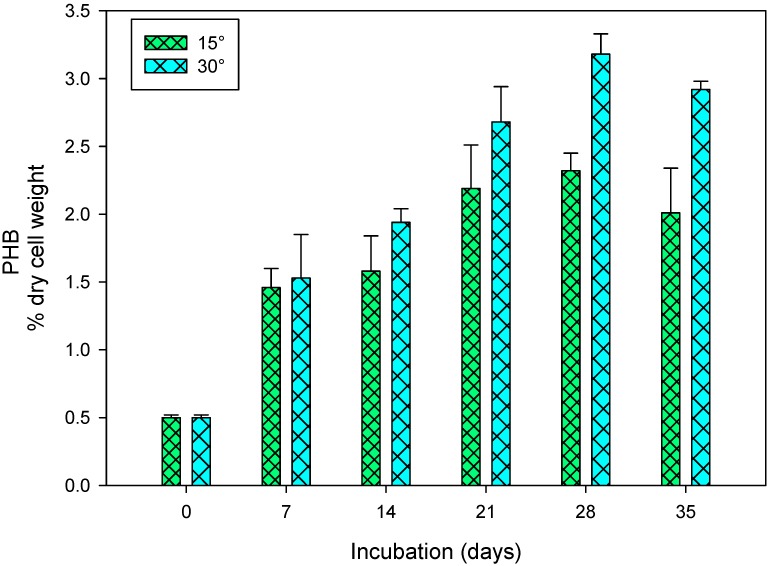
Production of PHB in *S. geitleri* in response to temperature. Values are the mean (*n* = 3) ± SD.

The pH and temperature are the key factors affecting the cyanobacterial metabolism. To ascertain the response of pH and temperature on PHB production in *S. geitleri*, the cells of *S. geitleri* were grown at different pH (5.5–10.5) and temperatures (15 and 30 °C) for 7–35 days and PHB production was measured (Figure 5 and Figure 6). Sodium acetate (50 mM, pH 5.5), potassium phosphate (50 mM, pH 7.5) and glycine-NaOH (50 mM, pH 8.5–10.5) buffers were used to maintain the different pH. It is evident from the data of Figure 5 that the cells of *S. geitleri* grown at pH 8.5 produced the maximum amount of PHB (3.98% of dry cell wt) on the 28th day of incubation. Further, an increase or decrease in pH from pH 8.5 resulted in a significant decrease in PHB production. At pH 8.5, the maximum PHB accumulations were also recorded in *Synechocystis* sp. PCC6803 [35]. Ansari and Fatma [29] also found similar results and suggested that alkaline pH (8–9.5) favored high PHB accumulation compared to acidic pH (5–7). Likewise, *S. geitleri* grown at 30 °C produced higher amounts of PHB (3.18% cell dry wt.) compared to that grown at 15 °C (Figure 6). The cells of *S. geitleri*, however, produced an equal amount of PHB on the seventh day at both the temperatures. *Nostoc muscorum* NCCU-442 also accumulated maximum PHB at 30 °C when compared to 15, 20, 25, 40, 45 °C [29]. In case of *Synechocystis* sp. PCC6803, maximum PHB accumulation occurred at 28 °C [35]. It is a well-established fact that cyanobacteria grows well at relatively high temperatures (25–27 °C) and in alkaline conditions. Furthermore, enzyme activity proceeds with a maximum rate at optimum temperatures which varies with organisms. In the present study, the ability of *S. geitleri* to produce maximum PHB at pH 8.5 and 30 °C was also supported by the findings of other workers [44]. Karbasi et al. [45] also studied the PHB production in four different bacterial species, viz. *Azotobacter beijinckii* DSMZ 1041, *Cupriavidus necator* DSMZ 545, *Hydrogenophaga pseudoflava* DSMZ 1034, and *Azohydromonas lata* DSMZ 1123 and found that maximum PHB accumulation occurred at 30 °C.

## 4. Conclusions

Cyanobacterium *S. geitleri* produced high quality (in the form of tough, translucent and grey film) PHB. The PHB production showed a linear relationship with the growth pattern of the cyanobacterium. The *S. geitleri* physiologically adapted to varying environmental conditions and produced maximum PHB at pH 8.5 and 30 °C temperatures, and acetate (30 mM) was the preferred carbon source. Compared to other microbial sources, cyanobacterial PHB is supposed to be economical and can be used as an alternative for biodegradable bioplastics on a large scale. In the current scenario of increasing pollution loads, *S. geitleri* explores a new alternative of conventional petrochemical polymers to reduce microplastics pollution and marine littering.
